# Beyond muscle weakness: pathogenesis of sepsis-induced myopathy and its management

**DOI:** 10.1515/med-2025-1342

**Published:** 2026-03-06

**Authors:** Yukun Liu, Xuan Zhao, Zhikai Xu, Qinxin Liu, Yuchang Wang

**Affiliations:** Department of Plastic and Aesthetic Surgery, Tongji Hospital, Tongji Medical College, Huazhong University of Science and Technology, Wuhan, China; Division of Trauma Surgery, Emergency Surgery & Surgical Critical, Tongji Hospital, Tongji Medical College, Huazhong University of Science and Technology, Wuhan, China; Trauma Center, Tongji Hospital, Tongji Medical College, Huazhong University of Science and Technology, Wuhan, China; Department of Emergency and Critical Care Medicine, Tongji Hospital, Tongji Medical College, Huazhong University of Science and Technology, Wuhan, China; Sino-German Research Institute of Disaster Medicine, Wuhan, China

**Keywords:** sepsis-induced myopathy, sepsis, muscle wasting, muscle atrophy, ICUAW

## Abstract

**Background:**

Sepsis-induced myopathy (SIM)significantly contributes to long-term disability and mortality among sepsis survivors. A comprehensive understanding of both the molecular mechanisms and rehabilitation strategies is crucial for effective management.

**Methods:**

A review of pertinent studies was conducted, focusing on the molecular pathogenesis, therapeutic strategies, and rehabilitation interventions for SIM, with particular attention to clinical and translational advancements.

**Results:**

Current management strategies encompass infection control, modulation of inflammation, nutritional support, and structured rehabilitation programs, including early mobilization and physiotherapy. Emerging therapies that target inflammation, cellular protection, and regeneration – such as stem cell therapy and gene-editing techniques – demonstrate potential. Furthermore, advancements in personalized medicine, including genomics, transcriptomics, and individualized metabolic interventions, may further improve outcomes.

**Conclusions:**

Optimizing both mechanistic and rehabilitation strategies is essential for enhancing functional recovery and quality of life in patients with SIM. An integrated clinical and molecular approach presents the most promising path forward. Keywords: sepsis-induced myopathy, sepsis.

## Introduction

The notion of sepsis-induced myopathy (SIM) was initially introduced by Professor Leigh Ann Callahan of the University of Kentucky in 2009(1) and is alternatively known as intensive care unit-acquired weakness (ICU-AW) [1], 2].SIM is a myopathy resulting from sepsis, predominantly impacting limb muscles, respiratory muscles, and the diaphragm, leading to decreased muscle strength, muscle atrophy, and changes in the biological characteristics of muscle [3], 4]. This leads to diminished motor ability and muscle strength, restricted functional activity, prolonged mechanical breathing, challenges in weaning from ventilators, illness exacerbation, protracted hospitalization, and elevated patient mortality [5], [6], [7], [8], [9], [10].

Skeletal muscle is essential for systemic homeostasis, functioning as the primary organ for voluntary movement but also as a major reservoir for amino acids and energy substrates during stress situations [11], 12]. Muscle tissue contributes to glucose metabolism, lipid oxidation, and endocrine signaling via the release of myokines, which influence immune responses and organ function. Skeletal muscle consists of different fiber types, including type I (slow-twitch, oxidative), type IIa (fast-twitch, oxidative-glycolytic), and type IIb/x (fast-twitch, glycolytic) fibers, each exhibiting unique metabolic and contractile properties [13]. Type I fibers are fatigue-resistant and rely primarily on oxidative phosphorylation, whereas type II fibers generate rapid force but are more susceptible to fatigue and metabolic stress. Emerging evidence suggests that different muscle fiber types exhibit heterogeneous responses to sepsis [14]. Type II glycolytic fibers seem more susceptible to sepsis-induced atrophy and proteolysis, likely due to higher basal rates of protein turnover and diminished oxidative capacity, whereas type I fibers may retain function longer but still undergo structural and metabolic alterations under prolonged inflammatory stress [15]. Comprehending these fiber-specific susceptibilities is essential for formulating focused strategies to maintain muscle mass and functionality in individuals with sepsis.

The primary cause of sepsis-induced muscle atrophy is the imbalance between reduced muscle protein synthesis and increased protein degradation, leading to a severe disruption of anabolic and catabolic processes [16], [17], [18], [19], [20], [21]. Initial research indicated that heightened protein breakdown was predominantly facilitated by the activation of mechanisms including the ubiquitin-proteasome system (UPS) and autophagy [22], [23], [24], [25], [26], [27], [28], [29], [30]. Myofibrillar proteins are initially degraded by caspase activation and cleavage, followed by further degradation via the UPS pathway. Persistent inflammatory stimuli eventually lead to weight loss in affected patients [31], 32]. Excessive activation of autophagy leads to structural and functional mitochondrial malfunction in muscle cells, resulting in muscle weakening and atrophy [33], 34].

Regarding skeletal muscle anabolism, previous research has established that the phosphoinositide 3-kinase (PI3K)/serine-threonine kinase (AKT) signaling pathway facilitates muscle protein synthesis and suppresses protein breakdown. This transpires via the upregulation of the mammalian target of rapamycin (mTOR), which promotes muscle synthesis, and the inhibition of forkhead box protein O (FOXO), thereby downregulating the transcription of atrophy-related genes, such as muscle-specific RING finger protein 1 (MuRF1) and atrogin-1 (Atrogin1) [35], 36].

The existing treatment for SIM is primarily limited to infection management, inflammatory modulation, nutritional assistance, and rehabilitative therapy, but its efficacy remains inadequate. Recent research has uncovered a growing array of putative molecular pathways causing SIM, presenting more promising treatment options. Therefore, this review discusses the possible molecular mechanisms and therapeutic prospects of SIM, with the objective of offering more effective and personalized treatment options for patients, thus enhancing their recovery and quality of life.

## Molecular mechanisms of sepsis-induced muscle atrophy

### Ubiquitin-proteasome system

**Figure 1:**
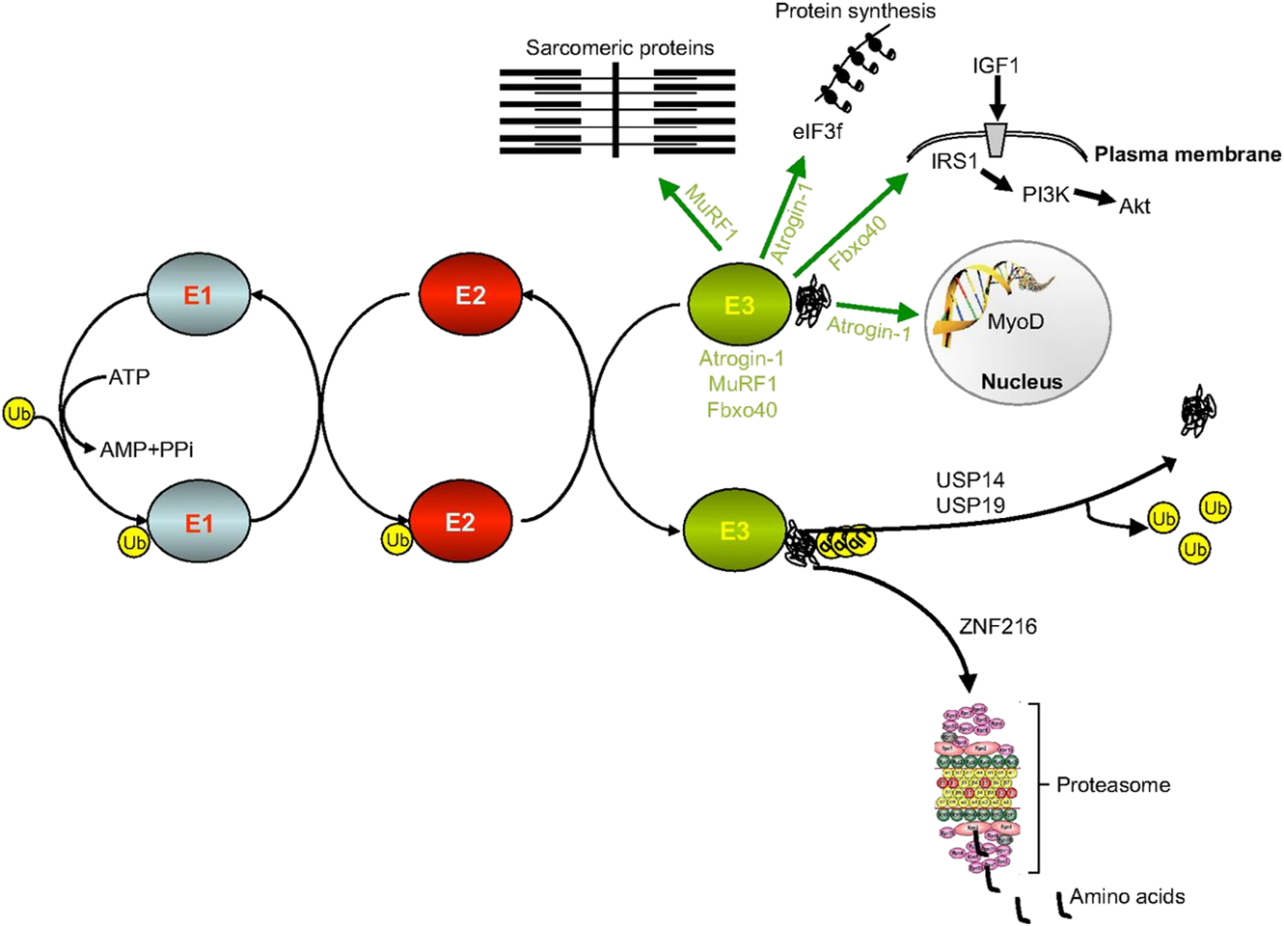
Covalent modification of protein substrates by ubiquitin via thioester cascade reactions. Ubiquitin-activating enzyme E1 activates ubiquitin (Ub, green circle) through ATP hydrolysis, forming a thioester bond and transferring the activated ubiquitin to the E2 ubiquitin-conjugating enzyme. Subsequently, E2 interacts with its specific E3 ubiquitin ligase, facilitating the covalent attachment of ubiquitin to protein substrates. This thioester cascade reaction must cycle multiple times to form a ubiquitin chain. Deubiquitinating enzymes (DUBs) antagonize this process to regulate protein degradation and recycle ubiquitin [37].

The ubiquitin-proteasome system (UPS) is an essential cytoplasmic protein degradation route in eukaryotic cells, integral to cell cycle regulation, apoptosis, inflammation, transcription, signal transmission, protein quality control, and various other biological functions. The UPS mechanism comprises a sequence of enzymes (E1, E2, and E3) that attach ubiquitin to lysine residues of target proteins through their C-terminus, a process termed ubiquitination [21], 31] (Figure 1. Ubiquitinated target proteins are identified by particular receptors (RP, also known as the 19 S regulatory particle or PA700) on the proteasome, which facilitates their translocation into the core particle (CP, sometimes referred to as the 20 S proteasome) for disintegration [38], [39], [40], [41]. Sarcomeres are composed of actin, myosin heavy chains, and titin, delineated by two Z-discs, with the M-line situated in the middle of the sarcomere between the Z-discs [42], 43]. MuRF1, an E3 ligase that conjugates active ubiquitin to lysine residues of target proteins, predominantly associates with the M-band through its interaction with titin; however, it is also located at the Z-disc [44], 45]. MuRF1 ubiquitinates and promotes the proteasomal degradation of myosin-binding protein C (MyBPC), myosin light chains one and 2 (MLC1/2), and troponin I3 (TNNI3) [46], 47]. MuRF2 is situated in the M-band and Z-disc and may engage with proteins linked with MuRF1. Research indicates that cardiomyocytes of MuRF1 knockout mice, which maintain MuRF2 expression, display hypertrophy, implying that the absence of MuRF1 interferes with protein breakdown mechanisms [48], 49]. MuRF3 is linked to the Z-disc and glutamylated microtubules [50], suggesting that MuRF proteins demonstrate specificity for sarcomeric targets.

Moreover, tripartite motif-containing protein 32 (TRIM32), an additional E3 ligase, is localized to the Z-disc and may facilitate the degradation of actin (ACTA1), tropomyosin (TMP1), troponin (TNNI3), α-actinin (ACTN1 and ACTN3), and Z-disc-associated intermyofibrillar proteins [51]. In a model of muscle atrophy induced by denervation, TRIM32 was identified as a degrader of desmin filaments, essential for preserving muscle morphology and function [52], 53]. Desmin filaments are crucial for muscle architecture and functionality, as they interconnect neighboring myofibrils at the Z-line and associate them with the sarcolemma, mitochondria, and nuclear membrane [53].

Significant regulators of Z-disc proteins comprise cullin-RING ligases (CRLs), with the prototype CRL being the SKP1-CUL1-F-box protein (SCF) complex, a modular assembly that facilitates specialized interactions with a diverse range of protein substrates through its fundamental SCF architecture [54]. The muscle-specific F-box protein atrogin-1/MAFbx is a pivotal regulator of cellular dimensions [55]. MAFbx, a CUL-1 adaptor, localizes to the sarcomeric Z-disc and ubiquitinates and degrades structural proteins, including α-actinin-2 (ACTN2), desmin (DES), and the regulatory protein calcineurin (CALNA) [56], 57]. Recently, deubiquitinating enzymes (DUBs), including USP2, UEP14, USP19, USP25, and USP28, have been recognized as regulators of the ubiquitin-proteasome system (UPS) in muscle tissue. USP19 has attracted considerable attention due to its upregulation during muscle atrophy and its potential role in regulating certain myofibrillar protein levels [58], 59]. These data collectively indicate that the UPS is crucial in the terminal degradation phase of muscle proteins.

### Autophagy

**Figure 2:**
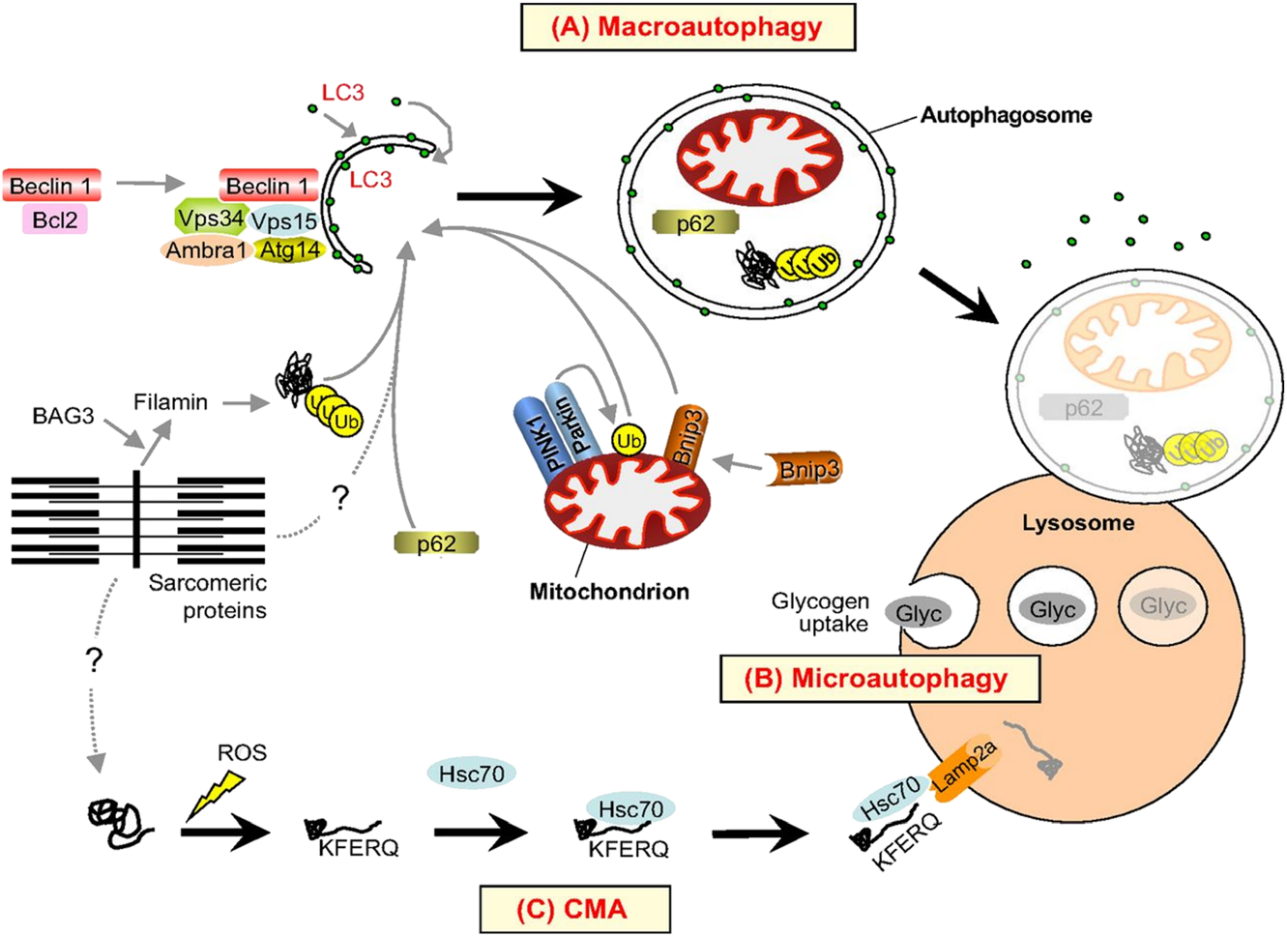
The role of macroautophagy, microautophagy, and chaperone-mediated autophagy (CMA) in protein degradation and organelle clearance in skeletal muscle. (A)Macroautophagy is initiated by a regulatory complex (including Vps34, Beclin 1, Vps15, Ambra1, and Atg14), which activates LC3 recruitment to the nascent autophagosome (isolation membrane). Selective mitochondrial clearance (mitophagy, a specific form of macroautophagy) requires the involvement of the PINK1-parkin complex and Bnip3 factors. Ubiquitin-tagged proteins (such as BAG3 and filamin) are delivered to the autophagosome by the scaffold protein p62, eventually undergoing lysosomal degradation. [1]. (B)Microautophagy involves the direct engulfment of small cytoplasmic components into the lysosome for degradation. Glycogen (Glyc) in skeletal muscle has been reported to be taken up and broken down through microautophagy. [12]. (C)Chaperone-mediated autophagy (CMA) primarily degrades proteins damaged by factors such as reactive oxygen species (ROS). These proteins expose specific amino acid sequences (KFERQ motifs), which are recognized by the Hsc70 chaperone protein and delivered to the lysosome for degradation via interaction with the Lamp2a receptor. [12]

Autophagy is a mechanism whereby segments of the cytoplasm or complete organelles are conveyed to lysosomes for destruction. Lysosomes, membrane-bound organelles containing diverse low-specificity proteases, facilitate the efficient destruction of most intracellular proteins delivered to them [38]. The physiological process of autophagy comprises three stages: (i) initiation, (ii) autophagosome creation, and (iii) lysosomal fusion and destruction [60], 61]. There are three forms of autophagy: microautophagy, chaperone-mediated autophagy (CMA), and macroautophagy [21], 62] (Figure 2).Microautophagy transpires in yeast and plants, involving the internalization of cytoplasmic debris by invagination of the lysosomal membrane [63]. CMA is a highly selective autophagic mechanism wherein intracellular proteins are directly translocated into lysosomes using molecular chaperones [64]. A defining characteristic of CMA is the transport of substrates to lysosomes via the chaperone Hsc70, a mechanism that operates independently of vesicles or membrane invagination, as the translocation complex on the lysosomal membrane enables the direct entry of substrates into the lysosomal lumen for degradation [64]. CMA substrates interact with LAMP2, a splice isoform situated on the cytosolic aspect of the lysosomal membrane, facilitating the assembly of the LAMP2 translocation complex [37], 65]. Macroautophagy entails the encapsulation of target proteins by autophagosomes, which subsequently merge with lysosomes for protein breakdown. Research indicates that only macroautophagy contributes to skeletal muscle atrophy [66]. In a lysosomal α-glucosidase deletion animal model, both type I and type II muscle fibers demonstrated enhanced autophagosome production [67]. Mitophagy, a distinct variant of autophagy, is governed by Parkin and PTEN-induced kinase 1 (PINK1) in mammals. Upon mitochondrial injury and loss of membrane potential, PINK1 accumulates in the cytoplasm and recruits Parkin to the dysfunctional mitochondria. Parkin ubiquitinates mitochondrial proteins, promoting their incorporation into autophagosomes for subsequent lysosomal destruction. This mechanism is regarded as a primary method for controlling mitochondrial amount in animals [68], [69], [70], [71], [72].

Research indicates that heightened mitophagy activity in denervated skeletal muscle atrophy models results in increased levels of reactive oxygen species (ROS), which aggravates muscle atrophy [73], 74]. A separate study revealed that in experimental models, autophagosomes were markedly elevated in gastrocnemius muscle fibers, accompanied by the upregulation of autophagy-initiating genes (UIK1, Pik3c3) and receptor-mediated mitophagy genes (Binp3 and Binp3I), leading to a substantial decrease in mitochondrial quantity and a significant reduction in skeletal muscle strength and mass [34], 75]. Apigenin, an antioxidant, has demonstrated efficacy in mitigating age-related muscle atrophy in mice by suppressing oxidative stress, mitophagy, and apoptosis in skeletal muscle, thereby drastically decreasing the expression of the autophagy marker LC3B in muscle fibers [76]. The apolipoprotein B mRNA editing enzyme catalytic polypeptide (APOBEC) family comprises cytidine deaminases, including Apobec2, which is only expressed in the differentiated cardiac and skeletal muscle of mammals and birds. Apobec2 deficiency results in enhanced mitophagy and a marked decrease in mitochondrial quantity in skeletal muscle [77]. Studies demonstrate that mitophagy-related functional decline and mitochondrial depletion are significant factors in skeletal muscle atrophy. The overexpression of mitochondrial E3 ubiquitin ligase 1 (Mul1) enhances mitophagy and muscle atrophy by promoting the ubiquitination and degradation of the mitochondrial fusion protein Mfn2, hence diminishing mitochondrial quantities [77], 78].

### Calpain system

The calpain system is part of the calcium-dependent cysteine proteases and is found in all mammals. It consists of three molecular components: two Ca2+-dependent proteases (μ-calpain and *m*-calpain) and calpastatin, which inhibits the activity of these two proteases [79]. In sepsis-induced muscle wasting, elevated calcium absorption in skeletal muscle activates calpains, resulting in the degradation of myofibrillar cytoskeletal proteins, disruption of sarcomere architecture, and release of myofilaments, which are then ubiquitinated and degraded by the 26 S proteasome. Moreover, calpains govern the degradation of transcription factors linked to muscle atrophy and may directly or indirectly affect 26 S proteasome activity. Furthermore, calpain activation diminishes Akt activity, thus increasing FoxO transcription factors and GSK-3β (which facilitate protein breakdown) while inhibiting mTOR signaling (which suppresses protein synthesis), thereby expediting muscle atrophy [31], 80]. A study revealed that calpains hydrolyze the Z-disc area of muscle fibers *in vitro*, resulting in their fragmentation into thick and thin filaments, which are then entirely destroyed in lysosomes. Furthermore, elevated Ca2+ concentrations markedly intensified the breakdown of muscle fibers, thus corroborating the synergistic influence of Ca2+ on the proteasome [81]. Moreover, calpains swiftly cleave troponin T/I, tropomyosin, and C protein, while their degradation rates for myosin and actin are significantly slower [82], [83], [84]. A separate investigation revealed that endotoxins markedly elevated calpain activity and the concentrations of activated μ-calpain and *m*-calpain in the diaphragm, concurrently diminishing diaphragm muscle strength [85].

### Acetylation and deacetylation modifications

Acetylation and deacetylation, encompassing transcription factors and nuclear coactivators, play a role in the control of muscle mass. Elevated acetylation levels may increase protein vulnerability to breakdown via multiple pathways, including enhanced intrinsic ubiquitin ligase activity mediated by histone acetyltransferases (HATs) [86]. Protein acetylation is as significant as phosphorylation and takes place on lysine residues. Lysine serves as a target for acetylation, ubiquitination, and sulfonation, providing fresh insights into the mechanisms of sepsis-induced muscle atrophy [87]. P300, a prominent histone acetyltransferase, is distinguished by its role in muscle cell atrophy and differentiation [88], [89], [90], [91], [92]. Research indicates that P300 facilitates the acetylation of histones and non-histone proteins, and is involved in the regulation of transcription factors and nuclear coactivators that govern muscle mass, including NF-κB/p65, FOXO transcription factors, C/EBPβ, and PGC-1α [93], [94], [95]. Sepsis can result in skeletal muscle atrophy, with both *in vivo* and *in vitro* studies indicating a considerable elevation in the expression and activity of HAT-P300 in muscle cells, and a reduction in the expression and activity of histone deacetylase-3/6 (HDAC3/6). As a result, the mRNA levels of MAFbx/atrogin-1 and MuRF1 were significantly increased, facilitating muscle fiber breakdown [89], 90], 92]. Glucocorticoids have been confirmed to stimulate muscle protein degradation and activate the ubiquitin-proteasome system. Upon stimulation of muscle cells with glucocorticoids, P300 protein levels exhibited an increase that was both time- and dose-dependent [89], 91].

### Oxidative stress and inflammation

**Figure 3:**
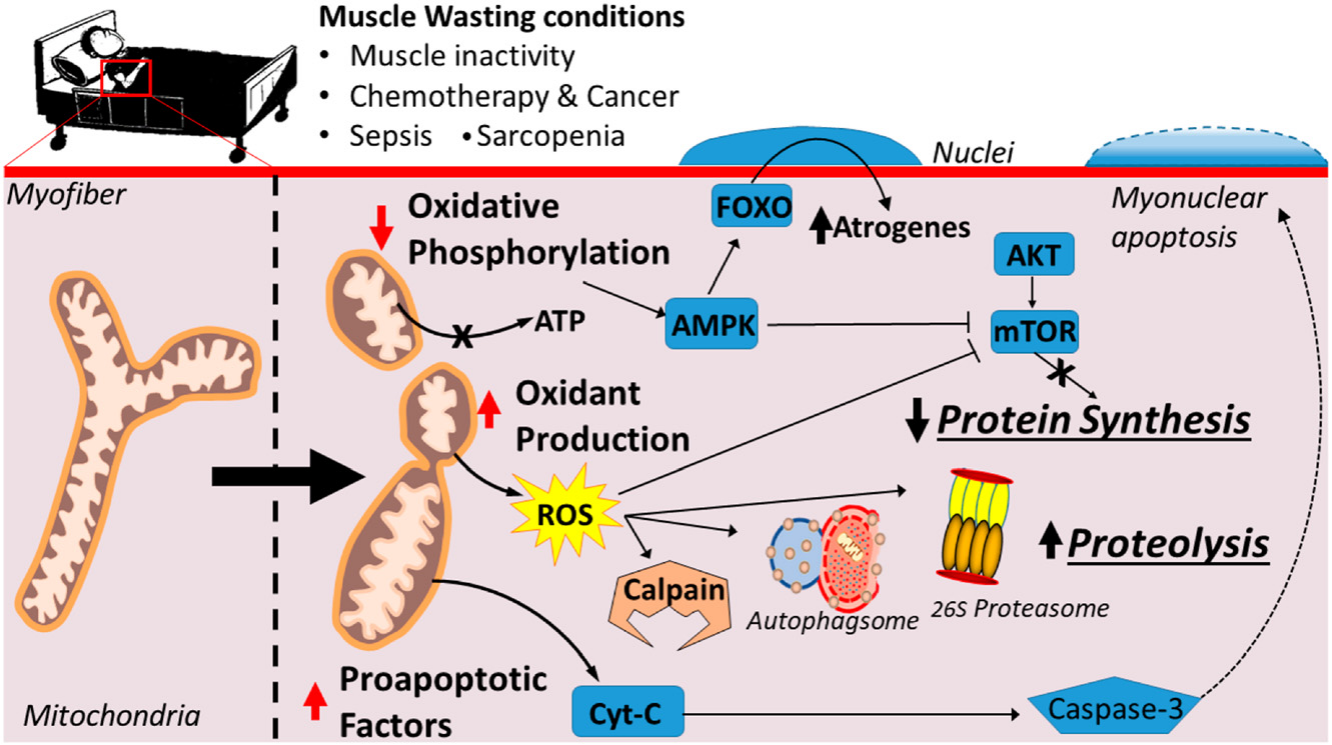
Mitochondrial dysfunction as a common denominator linking skeletal muscle wasting due to disease, aging, and prolonged Inactivity. Long-term muscle inactivity, aging, chemotherapy, cancer, and sepsis can lead to mitochondrial dysfunction, contributing to muscle wasting. Reduced mitochondrial respiratory capacity results in decreased ATP levels and activation of the AMPK signaling pathway. Elevated ROS levels activate major proteolytic pathways and inhibit muscle protein synthesis pathways. Additionally, mitochondria release pro-apoptotic factors, activating caspase-3 and mediating myonuclear apoptosis. [87].

Oxidative stress is seen as a significant pathogenic mechanism in SIM. During infection and inflammation, elevated reactive oxygen species (ROS) and oxidative stress agents cause intracellular oxidative damage, encompassing lipid peroxidation, protein oxidation, and DNA oxidation. Oxidative damage results in muscle cell dysfunction, structural degradation, and apoptosis. Moreover, oxidative stress can trigger inflammatory responses and the activation of inflammatory cells, creating a detrimental cycle that worsens muscle injury. Extended muscle inactivity and sepsis can result in mitochondrial malfunction, consequently inducing muscle atrophy. The reduction in mitochondrial respiratory ability leads to diminished ATP levels and the activation of the AMPK signaling system. Increased ROS levels can activate significant proteolytic mechanisms and impede muscle protein synthesis pathways. Additionally, mitochondria discharge pro-apoptotic substances, which activate caspase-3 and facilitate myonuclear apoptosis [96] (Figure 3).

In an inflammatory milieu, pro-inflammatory cytokines engage their specific receptors on the NF-κB, p38MAPK, and JAK/STAT pathways, facilitating muscle atrophy. Inflammation may indirectly provoke skeletal muscle atrophy by modifying the metabolic condition of other tissues or cells [97]. Diverse inflammatory factors and their subsequent pathways are regarded as potential targets for the treatment and prevention of skeletal muscle atrophy [31], 97]. The transcription factor nuclear factor-κB (NF-κB) constitutes a quintessential pro-inflammatory signaling pathway that enhances the expression of inflammatory mediators, such as cytokines, chemokines, and adhesion molecules, thereby playing a pivotal role in muscle atrophy [98]. Pro-inflammatory substances (e.g., TNF-α, IL-1) attach to their receptors, triggering NF-κB activation and the subsequent transcription of target genes, which influence the UPS to promote proteolysis and skeletal muscle atrophy. Thus, NF-κB is regarded as a crucial regulator and therapeutic target for muscle atrophy [98]. Triptolide inhibits LPS-induced skeletal muscle atrophy by obstructing the NF-κB/TNF-α pathway and regulating protein production and degradation pathways [99].

Increased plasma interleukin-6 (IL-6) concentrations are a risk factor for ICU-acquired weakness (ICUAW). Both *in vivo* and *in vitro* investigations have demonstrated that IL-6 facilitates sepsis-induced muscle atrophy via the gp130/JAK2/STAT3 signaling system [100]. IL-6 deficiency may mitigate skeletal muscle atrophy in septic rats by enhancing PGC-1α expression and suppressing mitochondrial ROS generation [101]. Moreover, IL-6 receptor antibodies proficiently mitigate muscle atrophy in sepsis and cancer cachexia by regulating the proteolytic system [102]. Localized injection of IGF-I inhibited sepsis-induced elevations in muscle IL-6 mRNA levels. Consequently, muscle-targeted IGF-I substantially alleviates sepsis-induced atrophy by augmenting muscle protein synthesis and possibly diminishing proteolysis.

### FOXO

Forkhead box O (FOXO) transcription factors are regarded as pivotal regulators of muscle atrophy in circumstances like sepsis, primarily due to their function in enhancing the expression of ubiquitin ligases atrogin-1 and MuRF1. In mammals, FOXO proteins are classified into FOXO1 (FKHR), FOXO3 (FKHRL1), and FOXO4 (AFX) [103]. At basal levels, FOXO is essential in suppressing satellite cell activation and constraining muscle development [104]. Research indicates that in septic rat muscle, the mRNA and protein levels of FOXO1 and FOXO3a elevate, whereas FOXO4 expression remains constant. Nuclear FOXO1 levels and DNA-binding activity are increased in septic muscle, but nuclear FOXO3a levels remain unchanged, indicating that the upregulation of atrogin-1 and MuRF1 in skeletal muscle is governed by FOXO1 [105]. In LPS-induced endotoxemia, the Akt/FOXO pathway may contribute to both diminished glucose oxidation at the PDC level and heightened muscle protein degradation [106]. Consequently, FOXO is considered a possible target for alleviating skeletal muscle atrophy [106]. A study showed that sulforaphane (SFN) promotes protein synthesis via Akt activation and mitigates dexamethasone-induced atrophy in C2C12 myotubes through a FOXO-dependent mechanism [107].

### Caspase-related cell death

Cysteine-aspartic proteases (caspases) constitute a family of proteases that are essential in regulating apoptosis, pyroptosis, and necroptosis [108]. Apoptosis is activated via two pathways [16]: the extrinsic pathway, which is dependent on caspase-8 activation, and [1] the intrinsic pathway, which is dependent on caspase-9 activation. Both pathways ultimately induce apoptosis by activating the effector caspases, specifically caspase-3, -6, and -7. In sepsis, lipopolysaccharide (LPS) regulates and activates the PLK1-AKT pathway, boosting myofiber death and decreasing myoblast growth, which ultimately results in skeletal muscle atrophy [109]. The targeted inhibition of caspase-3 and -7 or the initiator caspase-9, in skeletal muscle, has been shown to repair apoptotic synaptic degeneration in slow-channel congenital myasthenic syndrome [110]. Du and colleagues were the pioneers in recognizing that under catabolic conditions, the initial event precipitating muscle protein breakdown is the activation of caspase-3, which produces proteins designated for degradation by the ubiquitin-proteasome (Ub-P’some) system [32]. Research indicates that diaphragmatic atrophy is intensified by elevated caspase-3 activity, and this phenomenon can be mitigated by pretreatment with caspase inhibitors [111]. Caspase-3 may operate via two mechanisms. Initially, in catabolic conditions, caspase-3 is activated and cleaves muscle proteins, resulting in a distinctive 14-kDa actin fragment and additional substrates necessary for the ubiquitin-proteasome system (UPS). Secondly, caspase-3 activity may augment UPS-mediated protein degradation in muscle by directly boosting proteasomal proteolytic activity [112], 113]. Conversely, another study demonstrated that the deletion of caspase-3 safeguards against denervation-induced muscle atrophy via reducing apoptosis rather than through ubiquitin-proteasome-mediated protein degradation [114].

**Figure 4:**
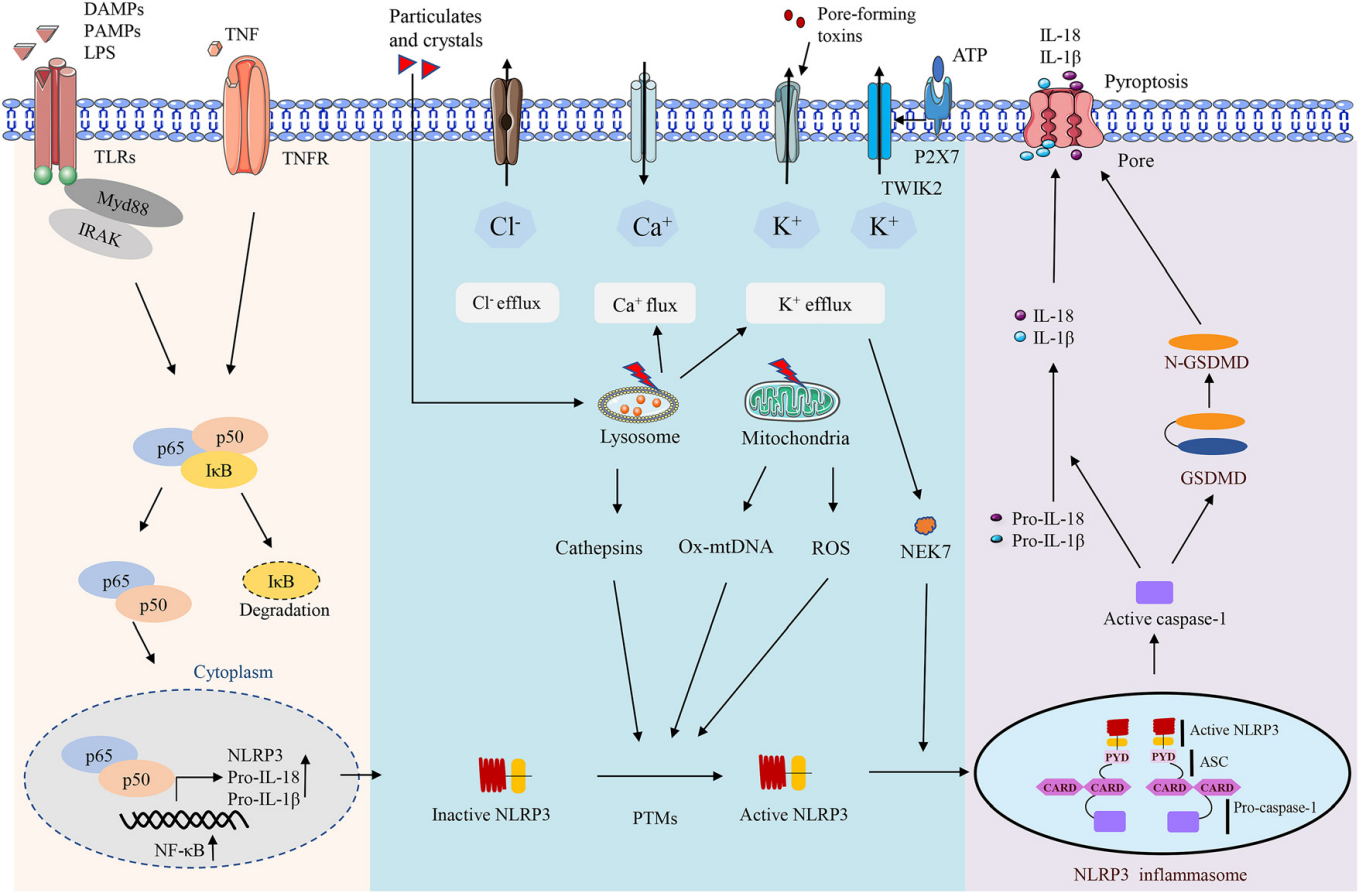
s intracellular infection and damage signals, triggering an inflammatory respons. Upon activation of the inflammasome, Caspase-1 is activated and further processes the pro-inflammatory cytokines IL-1β and IL-18, initiating an inflammatory reaction. Studies have shown that in sepsis-induced skeletal muscle atrophy, the activity and expression levels of Caspase-1 are significantly increased, and the release of its mediated inflammatory cytokines contributes to the inflammatory response and damage of skeletal muscle tissue. [107].

Pyroptosis, on the other hand, is mediated via both the classical caspase-1 pathway, which involves inflammasome activation, and the non-classical caspase-11/4/5 pathway [115]. Caspase-1 is a key component of the inflammasome, a multiprotein complex that detects intracellular infections and damage signals, thereby initiating inflammatory responses [116], [117], [118]. Upon inflammasome activation, caspase-1 is activated and subsequently processes the pro-inflammatory cytokines IL-1β and IL-18 into their active forms, triggering an inflammatory response [118], [119], [120], [121]. Studies have demonstrated that in sepsis-induced skeletal muscle atrophy, caspase-1 activity and expression are significantly upregulated, leading to the cleavage of gastrin D (GSDMD) into N-GSDMD, which forms pores in the cell membrane, ultimately causing pyroptotic cell death. The associated release of inflammatory cytokines further contributes to skeletal muscle inflammation and damage [122]. Inflammasome activation and its downstream effectors, including caspase-1, IL-1β, and IL-18, have been recognized as potential therapeutic targets for skeletal muscle atrophy [116], 123] (Figure 4). Recent studies have shown that selenomethionine attenuates sepsis-induced muscle atrophy by regulating the ROS/NLRP3 pathway, exerting antioxidant effects, and suppressing both inflammatory cytokines and pyroptosis, suggesting its potential as a therapeutic strategy for sepsis-associated muscle injury [124]. In addition, the P2X7 receptor has been demonstrated to drive sepsis-induced muscle atrophy through activation of NLRP3 inflammasome–mediated inflammation and pyroptosis; both genetic deletion and pharmacological inhibition of P2X7 significantly alleviate muscle damage, highlighting P2X7 as a promising therapeutic target [125].

### Mitochondrial dysfunction

The buildup of defective and impaired mitochondria in skeletal muscle is regarded as a crucial contributor to the onset of muscular weakness during sepsis [16], 126]. Mitochondrial dysfunction in skeletal muscle has also been recognized as a primary factor in ICUAW [127]. Under physiological settings, mitochondria continuously undergo fission and fusion, creating a dynamic interconnected network that meticulously manages mitochondrial architecture, size, and abundance [128], 129]. In septic muscle, an elevation in mitochondrial volume and complexity may arise from diminished DRP1 expression and activation, potentially disturbing the equilibrium between fusion and fission, resulting in excessive mitochondrial fusion [128].

A recent study indicated that muscle-specific deletion of DRP results in significant muscle impairment, marked by atrophy, weakness, fiber degradation, and mitochondrial dysfunction [130]. In a cecal ligation and puncture (CLP)-induced sepsis paradigm, sepsis resulted in skeletal muscle dysfunction, correlated with reduced expression of mitochondrial calcium uptake protein 1 (MICU1) in skeletal muscle mitochondria [131]. Mitochondrial and metabolic changes have been associated with sepsis-induced satellite cell dysfunction and compromised muscle regeneration. Transplantation of mesenchymal stem cells has been demonstrated to diminish cytokine levels, restore mitochondrial and metabolic activity in satellite cells, and enhance muscle strength [132].

Recent research has further established the causal relationship between mitochondrial abnormalities and chronic post-sepsis muscular weakening. In a mouse model of severe sepsis with ICU-like therapies, researchers discovered that sepsis survivors demonstrated enduring significant muscle weakness for a minimum of one month, despite recovering muscle mass. This was linked to aberrant mitochondrial ultrastructure, compromised mitochondrial respiration and electron transport chain function, and persistent protein oxidative damage in skeletal muscle [133]. Significantly, transgenic mice that overexpress the mitochondria-specific antioxidant enzyme MnSOD exhibited protection against mitochondrial abnormalities and muscle weakness, whereas pharmacological intervention with the mitochondria-targeting tetrapeptide SS-31 during sepsis effectively averted the subsequent onset of muscle weakness [134]. The findings suggest that ongoing mitochondrial dysfunction, rather than solely muscular atrophy, significantly contributes to chronic muscle weakness following sepsis, underscoring the potential of mitochondria-targeted therapy to enhance functional recovery in sepsis survivors.

## Therapeutic strategies

**Figure 5:**
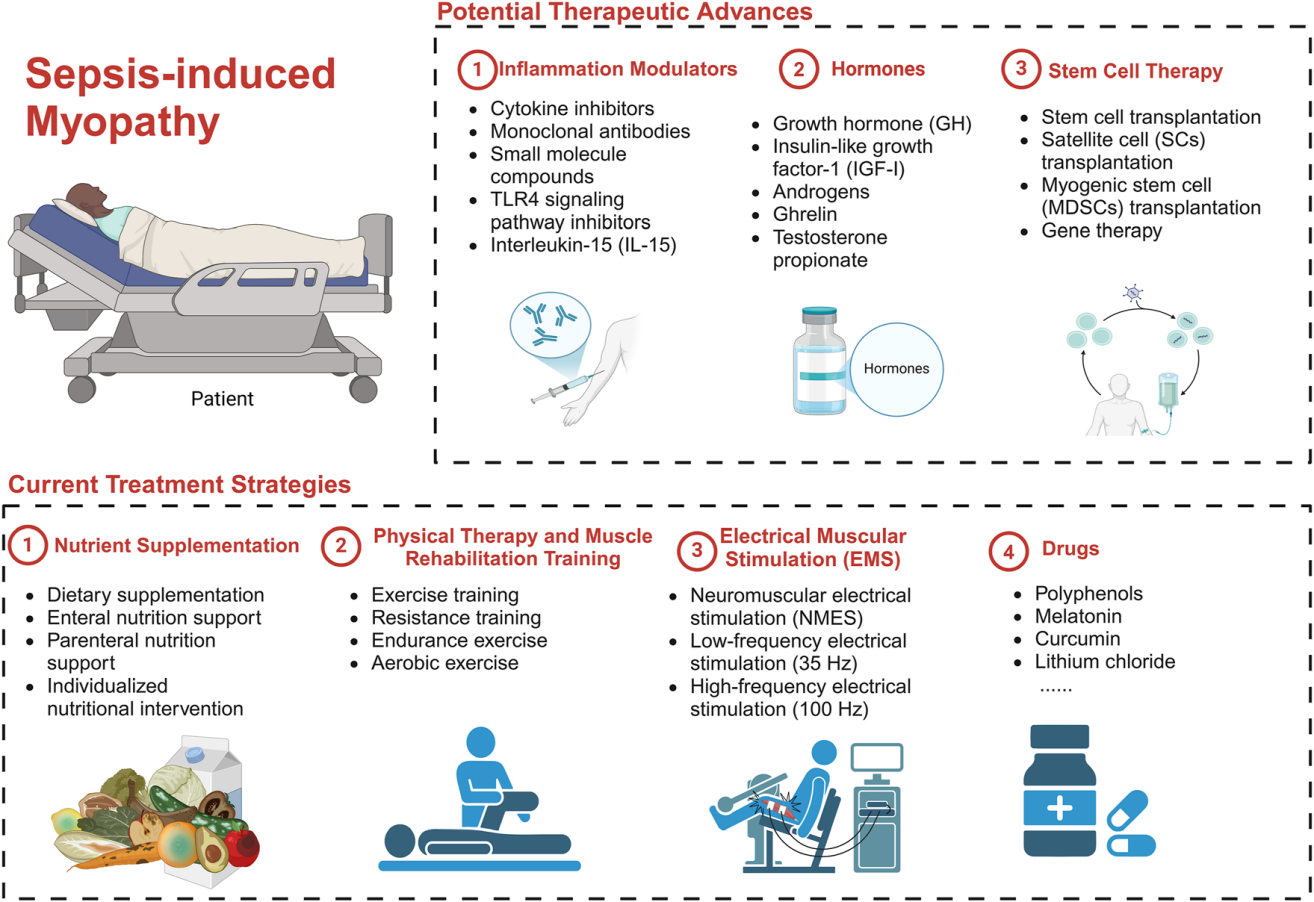
SIM treatment strategies encompass a range of approaches aimed at preventing and alleviating muscle wasting. Nutritional supplementation, including protein and energy intake through dietary, enteral, and parenteral methods, is crucial for maintaining muscle function. Physical therapy and muscle rehabilitation, such as exercise, resistance training, and electrical muscle stimulation (EMS), have been shown to enhance muscle strength and mass. Pharmacological interventions, including polyphenols, melatonin, curcumin, lithium chloride, and β-adrenergic agonists, offer muscle-protective effects by modulating inflammation, oxidative stress, and muscle metabolism. These therapeutic strategies provide a comprehensive approach to managing SIM, though further clinical research is necessary to confirm their efficacy. Potential treatments for SIM include anti-inflammatory agents, hormonal therapies, stem cell treatments, and gene editing. Monoclonal antibodies and small molecule inhibitors targeting cytokines, like TAK-242, show promise in reducing inflammation and muscle atrophy. Hormonal treatments such as GH, IGF-I, and androgens can aid muscle recovery, though risks like insulin resistance need consideration. Stem cell therapy, particularly using satellite cells (SCs) and mesodermal stem cells (MDSCs), offers potential for muscle repair. Additionally, gene editing technologies like CRISPR may correct genetic defects, but further research is needed to validate these approaches. Created in https://BioRender.com.

Now, there is no standardized and efficacious treatment for SIM. Clinical management predominantly encompasses nutritional assistance, exercise rehabilitation, electrical stimulation, and pharmaceutical interventions. Furthermore, novel preclinical strategies like immunomodulation, stem cell therapy, and gene therapy have been investigated to enhance muscle metabolism, diminish inflammation, and facilitate muscle regeneration. These ideas are encapsulated in Figure 5.

### Nutritional supplementation

Patients with SIM may endure significant metabolic dysregulation and catabolic wasting, rendering adequate protein and energy supplementation essential for preserving nutritional status and promoting muscular function recovery [135]. Dietary supplements, enteral nutrition, and parenteral nutrition are prevalent methods, and personalized nutritional interventions can be customized according to the patient’s distinct requirements. Research indicates that dietary intervention can enhance the quality of life in individuals with muscular atrophy and expedite recovery from chronic illnesses [136]. Supplementation with l-glutamine (Gln) and/or l-leucine (Leu) has been documented to diminish sepsis-induced muscle breakdown and enhance the expression of myogenic genes. Leucine supplementation alone seems to significantly enhance the preservation of muscle mass during sepsis [137].

Nevertheless, it is important to acknowledge that, despite numerous studies, nutritional supplementation alone has not demonstrated a significant enhancement in muscle mass or functional status in individuals with muscular atrophy [138]. Furthermore, enteral feeding products fortified with prebiotics, dietary fiber, and probiotics have shown significant promise in alleviating sepsis-related myopathy [4], 139]. Specific micronutrients, like vitamin D, may confer advantageous benefits [140]. However, it should be noted that although early enteral nutrition (EEN) is considered beneficial for ameliorating muscle atrophy in septic patients, systematic reviews have shown that its impact on mortality is not significant, and the quality of evidence is limited [141]. Consequently, additional study is required to determine the effectiveness of dietary treatments.

### Physical therapy and muscle rehabilitation training

Physical treatment and muscular rehabilitation training are crucial elements of healing for patients with SIM [142], [143], [144]. Exercise training has demonstrated feasibility and efficacy as a therapeutic intervention. Certain research indicates that exercise prompts a proportional augmentation in myonuclear content, resulting in muscle fiber growth [145], and is regarded as a method for improving muscle hypertrophy and regeneration [146]. Resistance training can stimulate skeletal muscle anabolism and augment satellite cell proliferation [147], 148]. Moreover, endurance exercise enhances mitochondrial function in skeletal muscle, likely attributable to elevated PGC-1α expression [149]. Aerobic exercise has been shown to alleviate muscle atrophy by diminishing inflammatory reactions, limiting ubiquitin-proteasome activity [150], regulating inflammation [151], and preventing myocyte autophagy [152]. Early mobilization and rehabilitation training have shown potential in improving muscle function in septic patients, but their effects on long-term functional recovery remain controversial [153]. Therefore, it is important to note that not all patients are suitable for exercise therapy.

### Electrical muscular stimulation (EMS)

Electrical muscle stimulation (EMS) is extensively utilized in orthopedic and sports medicine for strength enhancement. Electrical muscle stimulation (EMS) has been regarded as a potential therapeutic intervention for patients suffering from ICUAW by inducing muscle contractions [154]. Clinical investigations have shown that neuromuscular electrical stimulation (NMES) effectively prevents muscle atrophy in critically ill patients with sepsis or septic shock in the ICU [154]. Nonetheless, prior research on the impact of EMS in septic patients has produced incongruous findings. Low-frequency (35 Hz) stimulation has been shown to be unsuccessful in maintaining muscle mass, while high-frequency (100 Hz) stimulation has been demonstrated to improve muscle strength [155], 156]. Research involving animals indicates that EMS may enhance muscle development while decreasing indicators of muscle atrophy and apoptosis [157]. Consequently, EMS demonstrates potential as an efficacious intervention for averting disuse muscle degeneration in ICU patients.

### Pharmacological interventions

Polyphenols have emerged as promising bioactive compounds that can reduce muscle atrophy caused by several pro-atrophic stimuli [158]. Several pharmacological agents with anti-inflammatory, free radical-scavenging, antioxidative stress, autophagy-lysosomal regulatory, and mitochondrial protective properties – such as melatonin [159], curcumin [160], lithium chloride [161], Dexmedetomidine [162], and the heme oxygenase-1 (HO-1) inducer hemin [163] – have shown protective effects in sepsis-induced multiple organ dysfunction. These medicines may also facilitate the repair and regeneration of skeletal muscle in SIM. β-adrenergic receptor agonists, which activate the β-adrenergic signaling pathway, have shown therapeutic potential by producing advantageous effects on skeletal muscle shape and function. Nonetheless, these constraints should not be disregarded [164]. Moreover, insulin resistance is a prevalent characteristic of sepsis and has considerable ramifications for protein and energy metabolism [165]. Impaired insulin signaling hinders glucose uptake and use in skeletal muscle, resulting in diminished anabolic signaling and increased proteolysis. This metabolic malfunction intensifies muscular atrophy and leads to sustained weakness in critically ill patients [166], 167]. A significant clinical contradiction is present in the management of SIM. Glucocorticoids, although advantageous for systemic inflammation and metabolic stabilization, can aggravate muscle atrophy by enhancing protein breakdown and disrupting anabolic pathways [168], 169]. This “therapeutic dilemma” underscores the difficulty of reconciling systemic therapy of sepsis with the maintenance of skeletal muscle development and function, highlighting the necessity for comprehensive treatments that address both organ protection and musculoskeletal function [170]. Due to the intricate pathophysiology of SIM, the pharmacological treatments have been verified solely in preclinical trials. Additional clinical trials are necessary to validate their efficacy and possible therapeutic uses.

### Consent statement

Not applicable. No individual personal data are included in the study.

## Potential therapeutic prospects

### Development and application of inflammatory regulators

Recent years have witnessed substantial advancements in the treatment of sepsis-related myopathy. Next-generation cytokine inhibitors are being developed to more accurately modulate the inflammatory response, diminish excessive cytokine release, and alleviate inflammatory damage to muscle tissues. Monoclonal antibodies directed against specific cytokines and tiny molecular inhibitors of cytokines have demonstrated potential in clinical trials, offering novel avenues for the therapy of sepsis-related myopathy. In both *in vitro* and *in vivo* sepsis models, the toll-like receptor 4 (TLR4)-specific signaling pathway inhibitor TAK-242 demonstrated efficacy in mitigating inflammation (including nuclear factor-κB activation, interleukin-6, and tumor necrosis factor-α expression) and reversing muscle atrophy induced by endotoxemia when employed for preconditioning cells or mice [171]. Interleukin-15 (IL-15) is regarded as a synthetic agent for skeletal muscle, which diminishes tissue cathepsin L activity and provides a protective effect against sepsis-induced muscle atrophy [172]. In addition, Triptolide mitigates LPS-induced muscle atrophy primarily by suppressing inflammatory cytokines such as TNF-α and IL-1β through NF-κB/NLRP3 inhibition, while enhancing IGF-1R/Akt/mTOR signaling [99].

### Hormones

Growth hormone (GH), insulin-like growth factor-I (IGF-I), and androgens are principal regulators of muscle metabolism in both health and disease, functioning as anabolic agents [173], 174]. Ghrelin, a 28-amino acid peptide predominantly secreted by the gut and hypothalamus (notably the stomach), serves as a natural ligand for the growth hormone secretagogue receptor (ghrelin receptor). It enhances food consumption, facilitates obesity, stimulates GH secretion, and assists in sustaining body weight and muscle mass by improving food absorption while modulating the expression levels of IGF-1 and GH(174). However, opposing viewpoints also exist in the literature [175]. It is crucial to acknowledge that pharmaceuticals aimed at IGF-1 or growth hormone levels may elevate the risk of illnesses such as diabetes or insulin resistance [176]. Moreover, testosterone propionate markedly improves skeletal muscular strength, endurance, and volume in septic rats, likely through the stimulation of the IGF-1/AKT pathway [177].

### Stem cell therapy

Stem cell therapy, as a novel treatment modality, is seen as having considerable potential for the repair and regeneration of injured muscle tissues. Stem cells possess the capacity for self-renewal and multipotent differentiation, allowing them to transform into muscle cells and contribute to tissue healing mechanisms. The effectiveness of myogenic stem cell transplantation in the treatment of muscular dystrophies has been validated [178]. Collins et al. [102] demonstrated that satellite cells, located beneath the basal lamina surrounding each muscle fiber, serve as myogenic precursors for muscle growth and repair. As few as seven satellite cells can merge with a transplanted muscle fiber to produce over 100 new muscle fibers, each containing thousands of myonuclei [179]. Moreover, transplanted satellite cells possess self-renewal capabilities, and their proliferation can replenish the host muscle. The functions of satellite cells (SCs) and mesenchymal-derived stem cells (MDSCs) in sarcopenia may facilitate the advancement of novel stem cell-based therapies for SIM [180]. Consequently, stem cell therapy presents potential as a novel treatment modality for sepsis-induced myopathy. Further investigation is required to resolve the safety and efficacy concerns related to stem cell therapies.

### Gene therapy and gene editing

Gene therapy and gene editing methodologies are a significant focus of rigorous investigation. Corrective genes can be introduced or aberrant genes edited to rectify genetic abnormalities in muscle cells and enhance their functionality [181]. In Duchenne muscular dystrophy (DMD), frame-shifting mutations in the dystrophin gene compromise muscle fiber integrity, resulting in muscle degeneration. AAV-Dmd CRISPR treatment partially ameliorated muscle function deficiencies and produced a cohort of endogenously corrected myogenic precursors in the musculature of mdx mice [182]. Alterations in the expression and functionality of specific nuclear cofactors, including histone acetyltransferase p300, histone deacetylases (HDACs) such as HDAC3, HDAC6, and SIRT1, together with nuclear cofactors PGC-1α and PGC-1β, facilitate muscle mass reduction under diverse catabolic situations [183]. Multiple studies indicate that diverse post-translational modifications (PTMs), including phosphorylation, acetylation, ubiquitination, SUMOylation, glycosylation, methylation, S-nitrosylation, carbonylation, and S-glutathionylation, play a role in the regulation of muscle health and illness. Multiple studies indicate that diverse post-translational modifications (PTMs), including phosphorylation, acetylation, ubiquitination, SUMOylation, glycosylation, methylation, S-nitrosylation, carbonylation, and S-glutathionylation, play a role in the regulation of muscle health and illness [184]. Therapeutic approaches aimed at transcription factors and nuclear cofactors may be formulated to avert and address muscle atrophy [185]. The advancement of these technologies offers renewed optimism for the treatment of sepsis-related myopathy; yet, obstacles persist in practical implementation, including efficient delivery mechanisms and the accuracy of gene editing.

The ongoing progress in genomics and transcriptomics technologies enables a more thorough comprehension of the genetic variants and gene expression traits associated with sepsis-related myopathy. Gene replacement therapy has the ability to rectify genetic abnormalities through the introduction of functional genes. Numerous promising therapeutic gene-mediated therapies are emerging, offering hope to individuals with spinal muscular atrophy and Duchenne muscular dystrophy [186], 187]. While no treatment now mitigates the progression of ALS, insights derived from SMA gene therapy have fostered optimism for the creation of effective and safe pharmaceuticals [188]. This personalized molecular diagnostic approach can help identify specific conditions and create individualized treatment plans. Patients with aberrant muscle protein metabolism may enhance the equilibrium of muscle synthesis and breakdown by protein metabolism modulators. The advancement of tailored therapies will rely on a comprehensive understanding of metabolic irregularities in sepsis-associated myopathy and accurate diagnostic instruments.

## Current challenges and future prospects

Investigations on sepsis-induced skeletal muscle atrophy (SIM) are scarce, with the majority of existing studies concentrating on established signaling pathways, including the ubiquitin-proteasome system, autophagy, and the PI3K/AKT/mTOR axis. Although these pathways are unequivocally implicated in the advancement of muscle atrophy during sepsis, they predominantly originate from animal models or *in vitro* research, which may not accurately reflect the intricate pathophysiology context in human patients. Thus, these mechanisms, while informative, do not comprehensively elucidate the variability of clinical presentations observed in septic patients, suggesting that supplementary factors – such as systemic inflammation, endocrine dysregulation, metabolic changes, microvascular dysfunction, and mitochondrial impairment – probably play a role in muscle wasting and functional deterioration.

At now, there is no unique or universally effective treatment for muscular atrophy triggered by sepsis. In clinical practice, management mostly involves supportive measures, such as infection control, nutritional supplementation, and early rehabilitation programs. These therapies can alleviate the advancement of muscle atrophy and partially maintain skeletal muscle strength. A considerable number of patients, especially those with extended ICU admissions or severe sepsis, suffer from enduring muscle weakness, chronic atrophy, or even irreversible paralysis, profoundly affecting their quality of life and functional autonomy. Moreover, the epidemiology of sepsis has evolved in recent years. The incidence of patients with polytrauma-induced sepsis, frequently arising from high-energy injuries, has been rising each year. These patients are especially susceptible to quick and severe skeletal muscle degradation resulting from systemic inflammatory responses, extended immobilization, and elevated catabolic stress. Comprehending the molecular foundations of sepsis-induced muscle atrophy in these patients is essential, not only for enhancing acute outcomes but also for promoting long-term rehabilitation and functional recovery.

Future research should focus on identifying new molecular and cellular pathways implicated in human SIM, particularly those associated with mitochondrial failure, compromised muscle protein synthesis, and microvascular deficiencies. Combining multi-omics methodologies – such as transcriptomics, proteomics, and metabolomics – with clinical data may reveal patient-specific susceptibility factors and prospective treatment targets, enhancing the understanding of disease causes.Moreover, formulating targeted strategies that integrate pharmaceutical treatments with individualized rehabilitation programs would be essential to avert irreversible muscle atrophy and enhance functional results. Investigating long-term recovery trajectories is crucial, as current research indicates that muscle weakness may linger despite the apparent resolution of atrophy, underscoring the necessity for extended monitoring and intervention measures. In summary, examining the mechanisms of sepsis-induced skeletal muscle atrophy is crucial for comprehending disease pathophysiology and has significant implications for formulating novel and effective treatment strategies, augmenting patient survival, and improving post-sepsis quality of life.

## Conclusions

Severe Immunosuppression in sepsis is a critical consequence characterized by inflammation, immunological dysregulation, oxidative stress, and mitochondrial dysfunction. Present therapies – primarily infection management, inflammatory regulation, and rehabilitation – provide restricted recuperation. Innovative techniques, such as mitochondria-protective medicines, cell-based regeneration, and precision medicine, have the potential to avert irreversible muscle weakening and enhance functional outcomes. Additional preclinical and clinical investigations are required to confirm their safety and efficacy.
